# A machine learning model incorporating the globulin-to-platelet index for predicting severe fibrosis in autoimmune hepatitis: A retrospective and prospective validation study

**DOI:** 10.1097/MD.0000000000048408

**Published:** 2026-05-08

**Authors:** Haiping Zhang, Jianping Ren, Xinming Li, Yinxue Ma, Lijuan Li, Chen Shao, Kechi Fang, Jie Lu, Huiping Yan, Yanmin Liu, Jing Wang

**Affiliations:** a Clinical Laboratory Center and Clinical Research Center for Autoimmune Liver Disease, Beijing Youan Hospital, Capital Medical University, Beijing, P. R. China; b EUROIMMUN Academy, Beijing, P. R. China; c State Key Laboratory of Cognitive Science and Mental Health, Institute of Psychology, Chinese Academy of Sciences, Beijing, China; d Department of Psychology, University of Chinese Academy of Sciences, Beijing, P.R. China; e Department of Pathology, Beijing Youan Hospital, Capital Medical University, Beijing, P. R. China; f Second Department of Liver Disease Center, Beijing Youan Hospital, Capital Medical University, Beijing, P. R. China.

**Keywords:** autoimmune hepatitis, globulin-to-platelet index, liver fibrosis, machine learning, noninvasive prediction, random forest

## Abstract

Accurate staging of liver fibrosis in autoimmune hepatitis (AIH) remains challenging due to the invasive nature and sampling limitations of liver biopsy. This study aimed to identify readily available predictors of severe fibrosis and to develop an AIH-specific noninvasive machine-learning model. This two-stage study retrospectively enrolled 208 patients with biopsy-confirmed AIH, with prospective validation in 26 additional patients. Transient elastography (TE) was performed in 110 retrospective and 12 prospective patients. Severe fibrosis was defined as Scheuer stages S3 to S4. Candidate variables underwent univariable and multivariable logistic regression with collinearity control. A random forest (RF) model was trained on the independent predictors and evaluated by the area under the receiver operating characteristic curve (AUROC), calibration, and decision curve analysis. Shapley Additive exPlanations were used for interpretability. Inflammatory activity was graded by Scheuer and prespecified for subgroup analyses (G0–G2 vs G3–G4). A TE-inclusive RF model was also developed in the TE subgroup. The globulin-to-platelet index, international normalized ratio, and blood urea nitrogen were identified as independent predictors of severe fibrosis in AIH. The RF model based on these variables yielded AUROCs of 0.863 (95% confidence interval [CI], 0.802–0.917) in the training set, 0.747 (95% CI, 0.602–0.863) in the test set, and 0.784 (95% CI, 0.556–0.959) in the prospective cohort. Stratified by inflammatory grade, AUROCs were 0.842 (95% CI, 0.757–0.914) in G0–G2 and 0.814 (95% CI, 0.726–0.886) in G3–G4. In contrast, the aspartate aminotransferase-to-platelet ratio index and fibrosis-4 index performed poorly overall and deteriorated further under moderate-to-severe inflammation. In the TE subgroup, the RF model outperformed TE alone (AUROC, 0.786 vs 0.682), and performance improved further when TE was integrated (AUROC, 0.898 [95% CI, 0.841–0.949]). Globulin-to-platelet index, international normalized ratio, and blood urea nitrogen were independent predictors of severe fibrosis in AIH. An RF model constructed from these markers provided a robust, noninvasive tool whose performance was preserved across inflammatory grades and was further enhanced by incorporating TE.

## 1. Introduction

Autoimmune hepatitis (AIH) is an inflammatory liver disease characterized by immune-mediated hepatocellular injury, elevated aminotransferases, hypergammaglobulinemia, and characteristic circulating autoantibodies.^[1]^ AIH affects individuals of all age groups but is more prevalent among females, with a male-to-female ratio of approximately 1:4 to 1:6.9.^[2]^ It is a chronic, progressive condition that may lead to cirrhosis and liver failure if not promptly recognized and adequately treated.^[3]^ Approximately one-third of adults with AIH have cirrhosis at diagnosis.^[4]^ Our previous work has also shown that 34.1% of anti-soluble liver antigen/liver-pancreas antibody-positive patients had end-stage liver disease, suggesting a more advanced clinical presentation in this seropositive subset.^[5]^ With ongoing hepatocellular injury, liver fibrosis may progress to cirrhosis both representing major contributors to liver-related mortality in AIH.^[6]^ Consequently, accurate assessment of liver fibrosis is critical for guiding treatment decisions, monitoring progression, and optimizing outcomes.^[1,7]^ However, liver biopsy, the reference standard for fibrosis staging, is limited by its invasive nature, sampling error (10–40%), costs, and risk of complications.^[8]^ Therefore, reliable noninvasive methods to assess liver fibrosis stage in AIH are urgently needed.

Existing noninvasive methods demonstrate significant limitations in AIH. The aspartate aminotransferase-to-platelet ratio index (APRI) and fibrosis-4 index (FIB-4), recommended by the World Health Organization for chronic hepatitis B (CHB),^[9–11]^ demonstrate poor diagnostic accuracy in AIH.^[12]^ Transient elastography (TE), which measures liver stiffness as a surrogate for fibrosis, generally outperforms these serum-based scores in AIH.^[13]^ However, TE readings can be confounded by inflammatory activity and are constrained by cost, equipment availability, and operator dependence.^[14,15]^ Consequently, robust AIH-specific blood-based tools remain scarce, particularly those incorporating novel serologic markers beyond conventional liver function tests.

Recent studies have proposed logistic regression-based nomograms for fibrosis prediction in AIH, including models by Zhang et al^[16]^ for severe fibrosis and Chen et al^[17]^ for significant fibrosis. Although these models show promising diagnostic performance, they rely on linear assumptions that may not fully capture the complex, nonlinear relationships inherent to AIH pathophysiology. Complementing regression, machine learning methods, particularly random forest (RF), can model nonlinearities and interactions among variables.^[18]^ In addition, composite indices such as the globulin-to-platelet index (GPI)^[19]^ may better reflect AIH-relevant biology and yield independent predictors not identified by conventional models.

This study aimed to identify independent predictors of severe fibrosis in AIH, and to develop a clinically applicable, AIH-specific, noninvasive RF model based on these predictors.

## 2. Materials and methods

### 2.1. Ethics approval

The study adhered to the Declaration of Helsinki and was approved by the Ethics Committee of Beijing Youan Hospital, Capital Medical University (No. [2012]44). Informed consent was waived, as this noninterventional study used only de-identified routine clinical data and posed minimal risk to participants.

### 2.2. Study design

#### 2.2.1. Inclusion criteria

Patients meeting criteria for probable or definite AIH according to the simplified International Autoimmune Hepatitis Group score (≥6).^[20]^ This system integrates autoantibody status, immunoglobulin G levels, histology, and exclusion of viral hepatitis; a score of 6 indicates probable AIH and ≥7 indicates definite AIH.

#### 2.2.2. Exclusion criteria

Comorbid viral hepatitis, Epstein–Barr virus infection, primary biliary cholangitis, hepatocellular carcinoma, or history of liver transplantation; absence of documented fibrosis staging unless cirrhosis was confirmed by imaging; aged <18 years; incomplete clinical or laboratory data.

**Figure 1.**
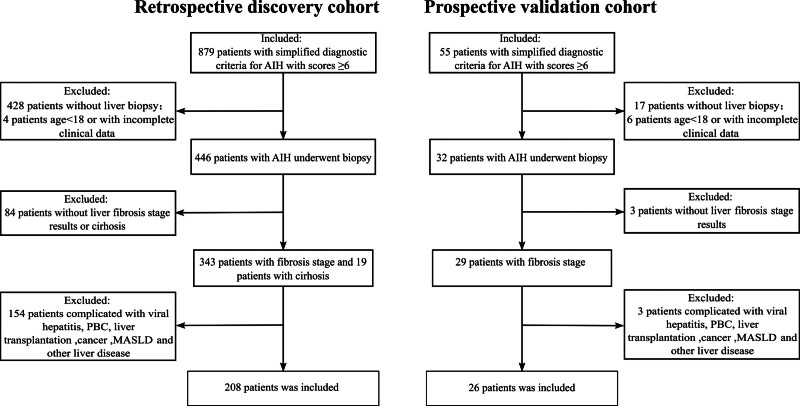
Flow chart of patient inclusion and exclusion criteria. AIH = autoimmune hepatitis, MASLD = metabolic dysfunction-associated steatotic liver disease, PBC = primary biliary cholangitis.

Initially, 879 patients fulfilling the inclusion criteria were retrospectively screened at Beijing Youan Hospital from July 2015 to March 2024. After applying the exclusion criteria, 208 patients constituted the retrospective cohort. A prospective validation cohort of 26 patients with complete data for all variables included in the final model was enrolled from August 2024 to November 2025. The patient selection process is shown in Figure 1.

### 2.3. Data collection

Electronic medical records were reviewed to obtain demographic and laboratory data within 2 weeks before liver biopsy or the diagnosis of cirrhosis. Only variables with fewer than 15% missing data were included in the analysis, unless they were important for assessing liver disease (including immunoglobulin G, immunoglobulin A, immunoglobulin M, alkaline phosphatase, gamma-glutamyl transferase (GGT), total bilirubin, and serum autoantibodies). For categorical variables, a dummy-variable approach was used, with missing values treated as a “missing” category. For continuous variables, missing values were imputed using the *k*-nearest neighbors algorithm (*k* = 5). Because autoantibodies are critical for AIH diagnosis and relatively stable, autoantibody results obtained closest to the biopsy were used for all patients. For the prospective validation cohort, data imputation was not performed.

The following variables were examined in this study: alanine aminotransferase, aspartate aminotransferase (AST), GGT, alkaline phosphatase, total protein, albumin, globulin, albumin-to-globulin ratio, prothrombin time (PT), international normalized ratio (INR), mean corpuscular hemoglobin (MCH), white blood cell count, hematocrit, red blood cell count, monocyte count and percentage, lymphocyte count and percentage, platelet count (PLT), estimated glomerular filtration rate, creatinine, blood urea nitrogen (BUN), total bilirubin, direct bilirubin, immunoglobulin G, immunoglobulin A, immunoglobulin M, and serum autoantibodies.

Indices and equations:


$$\begin{array}{l} APRI^{a}=\left(AST\left[U/L\right]/ULN^{b}\;for\;AST\right)/PLT\left(\times 10^{9}/L\right)\times 100 \end{array}$$

$$\begin{array}{l} \begin{array}{ll} FIB\text{-}4^{a}=\left(Age\left[years\right]\times AST\left[U/L\right]\right)\; & /\left(PLT\left[\times 10^{9}/L\right]\times {\left(alanine\;aminotransferase\left[U/L\right]\right)}^{1/2}\right)\; \end{array} \end{array}$$

$$\begin{array}{l} GPI=globulin\left(g/dL\right)\times 100/PLT\left(\times 10^{9}/L\right) \end{array}$$

^a^The optimal thresholds for APRI and FIB-4 were determined by receiver operating characteristic (ROC) curve analysis using Youden index on the overall cohort and applied across all subgroups in this study.

^b^ULN = upper limit of normal.

### 2.4. Liver histological assessment

This retrospective study reviewed the pathology records of patients who underwent liver biopsy. According to our institutional protocol for hepatitis evaluation, all biopsy specimens were routinely stained with hematoxylin and eosin (H&E), reticulin (silver impregnation), and Masson trichrome. Reticulin staining delineates the hepatic reticulin framework, which is essential for evaluating plate architecture and early parenchymal collapse, complementing Masson trichrome.^[21]^ Pathological reports were generated by 2 independent pathologists through consensus review at the time of initial diagnosis, and a senior pathologist (coauthor) subsequently verified data extraction. Liver fibrosis was staged according to the Scheuer scoring system^[22]^: S0, no fibrosis; S1, enlarged, fibrotic portal tracts without fibrous septa; S2, septal fibrosis with preserved architecture; S3, formation of fibrous septa with architectural distortion in the absence of cirrhosis; S4, cirrhosis. Imaging-confirmed cirrhosis was classified as S4. Severe liver fibrosis was defined as Scheuer stages S3 to S4.^[23,24]^ Inflammatory activity was graded using the Scheuer scoring system.^[22]^ In this study, Grade (G) 0 to G2 (indicating none, portal inflammation, or mild piecemeal necrosis) and G3 to G4 (indicating moderate to severe piecemeal necrosis) of liver inflammation were categorized as 2 distinct groups.^[25]^

### 2.5. TE measurement

TE (FibroScan; Echosens, Paris, France) was performed in 110 retrospective and 12 prospective patients (prospective TE data were excluded from TE-based validation). With the patient supine, breathing quietly, and the right arm elevated behind the head to maximize intercostal space, the probe was placed at the 7th to 9th intercostal space along the right mid-axillary line, and measurements were acquired during quiet respiration. Validity criteria were: ≥10 successful readings; success rate ≥60%; interquartile range-to-median (IQR/median) ratio ≤30%. The median of all valid readings was recorded as liver stiffness measurement (LSM, kPa). TE thresholds were derived by ROC analysis with Youden index in the retrospective TE subgroup and applied within its subgroups.

### 2.6. Variable screening for model construction

The retrospective cohort was randomly divided 7:3 into training and test sets; variable screening and model construction used only the training data. Variables were initially compared using *t* tests, *χ*^*2*^ tests, or Mann–Whitney *U* tests as appropriate, and those with *P* < .05 were entered into univariable logistic regression. Pairwise Pearson correlations were computed to avoid multicollinearity; when |*r*| > 0.7, the variable with the smaller absolute coefficient in univariable analysis was excluded. Remaining candidates were entered into multivariable logistic regression with backward stepwise elimination.

### 2.7. Random forest model development and appraisal

An RF model was trained to predict severe fibrosis using the independent predictors from Section 2.6. Model parameters were:


$$\begin{array}{lll} & n\_estimators=300,\;max\_depth\; & =3,\;n\_jobs=1,\;random\_state=0. \end{array}$$

Model generalizability was assessed in the test set and the prospective validation cohort. A TE-inclusive RF model was additionally trained in the TE subgroup by incorporating LSM alongside baseline features. Discrimination was assessed by ROC analysis; area under receiver operating characteristic curves (AUROC), sensitivity, specificity, accuracy, and F1 score were calculated. Calibration curves (bootstrap = 1000) and decision curve analyses were used to evaluate calibration and clinical applicability. SHapley Additive exPlanations (SHAP) were used to interpret feature contributions. For both RF models, which output predicted probabilities (0–1), we applied a default classification threshold of 0.5 consistently across all patient subgroups to ensure comparability. This threshold was used to compute sensitivity, specificity, accuracy, and F1 score for both model variants. Model discrimination was primarily evaluated using AUROC, which is independent of threshold selection.

### 2.8. Statistical analysis

The Kolmogorov–Smirnov test was used to assess the normality of continuous variables. Variables with normal distribution were presented as mean ± standard deviation and compared using independent samples *t* test. Continuous variables with non-normal distribution were presented as median (25th, 75th percentiles) and compared using Mann–Whitney *U* test. Categorical variables were reported as frequencies (percentages) and compared using the *χ*^*2*^ test or Fisher exact test. Differences between model AUROCs were assessed using DeLong test, and *P* values were adjusted using the Holm method for multiple comparisons. Autoantibody results were presented as the number positive among the total tested [n/N (%)]. Analyses were performed using IBM SPSS Statistics, version 29.0 (IBM Corp, Armonk) and Python 3.8 (Python Software Foundation). All tests were two-tailed, and *P* < .05 was considered statistically significant.

## 3. Results

### 3.1. Retrospective and prospective cohorts exhibited female predominance and high prevalence of severe fibrosis

A total of 208 retrospective and 26 prospective patients were included in this study (Fig. 1). Baseline characteristics were summarized in Table 1. The retrospective cohort served as the discovery cohort for model development, with a median age of 55 years and a male-to-female ratio of 1:7, consistent with the known epidemiology of AIH.^[26]^ According to Scheuer score criteria, fibrosis distribution was as follows: 41 patients (19.7%) in stage S0 to S1, 80 (38.5%) in S2, 57 (27.4%) in S3, and 30 (14.4%) in S4. Additionally, 50.5% of patients had inflammatory activity grades G3 to G4. Clinically significant complications included liver cirrhosis (n = 22), ascites (n = 10), hepatic encephalopathy (n = 2), and abdominal infection (n = 1). Among patients tested for autoantibodies, the antinuclear antibody positivity rate was 97.6% (202/207), anti-smooth muscle antibody positivity was 23.7% (49/207), and anti-soluble liver antigen/liver-pancreas antibody positivity was 7.0% (14/199).

**Table 1:** Baseline characteristics of participants in the retrospective discovery and prospective validation cohorts.

Variables	Retrospective cohort (n = 208)	Prospective cohort (n = 26)	*P* value
Female (%)	182 (87.5)	25 (96.15)	.327
Age (yr)	55.00 (47.00, 62.00)	58.50 (49.75, 64.25)	.344
ALT (U/L)	70.90 (35.15, 121.47)	61.50 (33.75, 120.50)	.602
AST (U/L)	97.00 (56.50, 170.31)	70.50 (42.50, 171.75)	.458
AST/ALT	1.41 (0.93, 2.13)	1.27 (0.73, 2.15)	.265
ALP (U/L)	118.50 (95.75, 153.50)	71.50 (41.25, 173.00)	.146
GGT (U/L)	121.30 (62.50, 200.18)	105.50 (82.50, 156.75)	.145
TP (g/L)	72.95 (57.23, 77.50)	70.80 (61.78, 74.53)	.062
Albumin (g/L)	35.53 ± 4.94	34.73 ± 4.83	.439
Globulin (g/L)	36.15 (32.58, 41.92)	34.30 (30.30, 40.25)	.063
A/G	0.98 ± 0.24	1.04 ± 0.27	.239
INR	1.12 (1.03, 1.20)	1.14 (1.04, 1.28)	.895
PT (S)	11.70 (10.50, 12.90)	11.05 (10.13, 12.80)	.207
MCH (pg)	31.55 (30.40, 32.92)	–	NA
WBC (×10^9^/L)	4.64 (3.71, 5.95)	4.15 (3.48, 6.26)	.584
Hematocrit (%)	36.10 (33.13, 39.80)	-	NA
RBC (×10^12^/L)	3.87 ± 0.55	3.75 ± 0.44	.291
Monocyte (×10^9^/L)	0.40 (0.31, 0.49)	0.34 (0.29, 0.47)	.208
Monocyte% (%)	8.25 (6.78, 9.80)	8.40 (6.85, 9.60)	.670
Lymphocyte (×10^9^/L)	1.69 (1.25, 2.22)	1.42 (1.21, 2.13)	.319
Lymphocyte% (%)	37.41 ± 11.61	35.05 ± 11.00	.326
PLT (×10^9^/L)	146.50 (107.00, 198.25)	127.50 (96.50, 177.25)	.583
eGFR (mL/min/1.73m^2^)	106.88 (100.08, 113.13)	104.55 (92.45, 111.35)	.198
Creatinine (μmol/L)	50.00 (44.00, 56.00)	56.00 (45.00, 61.00)	.275
BUN (mmol/L)	4.24 (3.64, 4.86)	4.55 (3.32, 5.67)	.357
DB (μmol/L)	18.00 (8.15, 35.36)	–	NA
TB (μmol/L)	30.60 (20.45, 51.45)	–	NA
Immunoglobulin A (g/L)	3.41 (2.82, 4.14)	2.92 (2.27, 4.45)	.125
Immunoglobulin M (g/L)	1.27 (0.98, 1.63)	0.92 (0.57, 1.65)	.729
Immunoglobulin G (g/L)	21.26 (18.59, 24.42)	21.30 (16.03, 26.88)	.529
GPI	2.52 (1.83, 3.59)	2.63 (1.78, 3.39)	.845
APRI	1.70 (0.86, 2.88)	1.70 (0.74, 3.31)	.824
FIB-4	4.31 (2.55, 7.80)	3.70 (2.62, 6.88)	.873
ANA (+)	202/207 (97.58)	26/26 (100.00)	1.000
ASMA (+)	49/207 (23.67)	6/25 (24.00)	.682
Anti-SLA/LP (+)	14/199 (7.04)	0/25 (0.00)	.376
Anti-LC-1 (+)	1/199 (0.50)	1/25 (4.00)	.211
Anti-LKM-1 (+)	1/207 (0.48)	1/26 (3.85)	.211
Fibrosis stage			.531
S0–S1	41 (19.71)	5 (19.23)	
S2	80 (38.46)	12 (46.15)	
S3	57 (27.40)	8 (30.77)	
S4	30 (14.42)	1 (3.85)	
Complications			
Liver cirrhosis	22 (10.58)	1 (3.85)	.484
Ascites	10 (4.81)	1 (3.85)	1.000
Hepatic encephalopathy	2 (0.96)	0 (0.00)	1.000
Intra-abdominal Infection	1 (0.48)	0 (0.00)	1.000
Inflammatory grade			.540
G0–G2	103 (49.52)	14 (56.00)*	
G3–G4	105 (50.48)	11 (44.00)*	

Note: – indicates data missing.

A/G = albumin-to-globulin ratio, ALP = alkaline phosphatase, ALT = alanine aminotransferase, ANA = antinuclear antibody, anti-LC-1 = anti-liver cytosol type 1 antibody, anti-LKM-1 = anti-liver kidney microsomal type 1 antibody, anti-SLA/LP = anti-soluble liver antigen/liver-pancreas antigen antibody, APRI = aspartate aminotransferase-to-platelet ratio index, ASMA = anti-smooth muscle antibody, AST = aspartate aminotransferase, BUN = blood urea nitrogen, DB = direct bilirubin, eGFR = estimated glomerular filtration rate, FIB-4 = fibrosis-4 index, GGT = gamma-glutamyl transferase, GPI = globulin-to-platelet index, INR = international normalized ratio, MCH = mean corpuscular hemoglobin, NA = not application, PLT = platelet count, PT = prothrombin time, RBC = red blood cell count, TB = total bilirubin, TP = total protein, WBC = white blood cell count.

* Data available for 25 patients.

The prospective cohort served as an external validation cohort, with a median age of 58.5 years and only 1 male patient. Fibrosis stage distribution was as follows: 5 patients (19.2%) in S0 to S1, 12 (46.2%) in S2, 8 (30.8%) in S3, and 1 (3.8%) in S4. No significant differences were observed in baseline characteristics between the retrospective and prospective cohorts.

For model development and internal validation, the retrospective discovery cohort was randomly divided into training and test sets at a 7:3 ratio; no significant differences in baseline characteristics were observed between these sets (see Table S1, Supplemental Digital Content, which compares baseline characteristics between training and test sets in the retrospective cohort).

### 3.2. Elevated GPI, INR, and BUN were identified as independent predictors of severe fibrosis on multivariable analysis

In the training set, 61 patients had severe liver fibrosis (Scheuer stages S3–S4, including cirrhosis) and 84 did not. Compared with the nonsevere fibrosis group, the severe fibrosis group exhibited higher levels of GPI, INR, PT, MCH, and BUN, while showing lower levels of GGT and PLT (all *P* < .05) (Table 2).

**Table 2:** Comparison of patients with severe and non-severe liver fibrosis in the training set.

Variables	Nonsevere fibrosis (n = 84)	Severe fibrosis (n = 61)	*P* value
Female (%)	76 (90.48)	49 (80.33)	.080
Age (yr)	53.50 (46.00, 61.00)	57.00 (48.00, 63.00)	.164
ALT (U/L)	75.00 (39.50, 129.22)	62.00 (29.90, 117.00)	.190
AST (U/L)	109.11 (57.00, 196.00)	83.00 (44.00, 150.00)	.105
AST/ALT	1.35 (0.86, 2.46)	1.36 (0.99, 2.03)	.898
ALP (U/L)	120.00 (90.23, 157.72)	115.00 (95.00, 148.00)	.649
GGT (U/L)	136.00 (77.47, 225.40)	97.00 (52.00, 159.00)	.023
TP (g/L)	72.29 ± 7.62	72.72 ± 8.19	.753
Albumin (g/L)	35.99 ± 4.73	34.79 ± 4.61	.130
Globulin (g/L)	35.70 (32.08, 40.35)	36.10 (32.60, 43.80)	.366
A/G	1.02 ± 0.22	0.96 ± 0.26	.155
INR	1.06 (1.01, 1.17)	1.14 (1.09, 1.24)	.002
PT (S)	11.38 ± 1.71	12.17 ± 1.99	.013
MCH (pg)	31.20 (30.25, 32.80)	32.20 (30.60, 33.40)	.048
WBC (×10^9^/L)	4.82 (3.77, 5.96)	5.27 (3.74, 6.52)	.740
Hematocrit (%)	36.11 ± 4.50	36.39 ± 4.78	.721
RBC (×10^12^/L)	3.88 ± 0.47	3.84 ± 0.52	.641
Monocyte (×10^9^/L)	0.41 ± 0.13	0.40 ± 0.17	.444
Monocyte% (%)	8.47 ± 2.45	7.84 ± 2.35	.118
Lymphocyte (×10^9^/L)	1.70 (1.37, 2.28)	1.66 (1.09, 2.22)	.203
Lymphocyte% (%)	38.53 ± 11.71	34.23 ± 11.80	.031
PLT (×10^9^/L)	179.38 ± 70.02	136.39 ± 56.66	<.001
eGFR (mL/min/1.73 m^2^)	108.03 (100.46, 113.71)	106.10 (98.68, 112.70)	.535
Creatinine (μmol/L)	50.46 (44.98, 56.25)	51.00 (44.00, 59.00)	.857
BUN (mmol/L)	4.16 (3.61, 4.74)	4.52 (3.74, 5.27)	.044
DB (μmol/L)	14.85 (8.28, 30.77)	19.06 (7.80, 35.40)	.681
TB (μmol/L)	26.05 (19.80, 42.43)	31.60 (21.70, 51.90)	.326
Immunoglobulin A (g/L)	3.29 (2.85, 4.08)	3.29 (2.65, 4.22)	.846
Immunoglobulin M (g/L)	1.23 (1.01, 1.61)	1.32 (0.93, 1.64)	.779
Immunoglobulin G (g/L)	21.00 (18.39, 23.70)	21.50 (19.00, 25.70)	.197
GPI	2.04 (1.59, 2.99)	2.92 (2.17, 3.99)	<.001
APRI	1.61 (0.78, 2.96)	1.66 (1.00, 2.86)	.561
FIB-4	3.79 (2.07, 7.50)	4.21 (2.76, 8.08)	.186
ANA (+)	81/84 (96.43)	61/61 (100.00)	.368
ASMA (+)	25/84 (29.76)	13/61 (21.31)	.253
Anti-SLA/LP (+)	4/78 (5.13)	6/61 (9.84)	.462
Anti-LC-1 (+)	0/84 (0.00)	0/61 (0.00)	NA
Anti-LKM-1 (+)	1/84 (1.19)	0/61 (0.00)	1.000
Inflammation			.101
G0–G2	46 (54.76)	25 (40.98)	
G3–G4	38 (45.24)	36 (59.01)	

A/G = albumin-to-globulin ratio, ALP = alkaline phosphatase, ALT = alanine aminotransferase, ANA = antinuclear antibody, anti-LC-1 = anti-liver cytosol type 1 antibody, anti-LKM-1 = anti-liver kidney microsomal type 1 antibody, anti-SLA/LP = anti-soluble liver antigen/liver-pancreas antigen antibody, APRI = aspartate aminotransferase-to-platelet ratio index, ASMA = anti-smooth muscle antibody, AST = aspartate aminotransferase, BUN = blood urea nitrogen, DB = direct bilirubin, eGFR = estimated glomerular filtration rate, FIB-4 = fibrosis-4 index, GGT = gamma-glutamyl transferase, GPI = globulin-to-platelet index, INR = international normalized ratio, MCH = mean corpuscular hemoglobin, NA = not application, PLT = platelet count, PT = prothrombin time, RBC = red blood cell count, TB = total bilirubin, TP = total protein, WBC = white blood cell count.

Univariable logistic regression analysis identified INR, PT, MCH, and BUN as associated with severe fibrosis (*P* < .05). Collinearity was observed between PT and INR (*R* > 0.7; *P* < .05) and between GPI and PLT (*R* > 0.7; *P* < .05). Because GPI and INR demonstrated higher absolute coefficients in univariable logistic regression, they were retained for multivariable logistic regression analysis along with other factors. Multivariable logistic regression identified GPI (odds ratio [OR], 1.370 [95% confidence interval [CI], 1.011–1.856]; *P* = .042), INR (OR, 37.523 [95% CI, 1.661–847.748]; *P* = .023), and BUN (OR, 1.491 [95% CI, 1.070–2.078]; *P* = .018) as independent predictors of severe fibrosis (Table 3).

**Table 3:** Univariable and multivariable logistic regression analysis of variables associated with severe liver fibrosis in the training set.

Variables	Univariable			Multivariable		
	Coefficient	OR (95% CI)	*P* value	Coefficient	OR (95% CI)	*P* value
GPI	0.459	1.583 (1.212–2.067)	.001	0.315	1.370 (1.011–1.856)	.042
MCH (pg)	0.159	1.172 (1.005–1.367)	.043	0.084	1.087 (0.913–1.295)	.347
Lymphocyte%	-0.032	0.969 (0.940–0.998)	.034	-0.032	0.969 (0.937–1.002)	.062
PLT (×10^9^/L)	-0.011	0.989 (0.984–0.995)	<.001			
INR	4.062	58.087 (4.058–831.513)	.003	3.625	37.523 (1.661–847.748)	.023
PT (s)	0.237	1.268 (1.050–1.531)	.014			
GGT (U/L)	-0.003	0.997 (0.994–1.000)	.075			
BUN (mmol/L)	0.328	1.388 (1.046–1.842)	.023	0.400	1.491 (1.070–2.078)	.018

BUN = blood urea nitrogen, CI = confidence interval, GGT = gamma-glutamyl transferase, GPI = globulin-to-platelet index, INR = international normalized ratio, MCH = mean corpuscular hemoglobin, OR = odds ratio, PLT = platelet count, PT = prothrombin time.

### 3.3. Random forest model achieved high predictive efficacy with GPI as the dominant predictor of severe fibrosis

**Figure 2.**
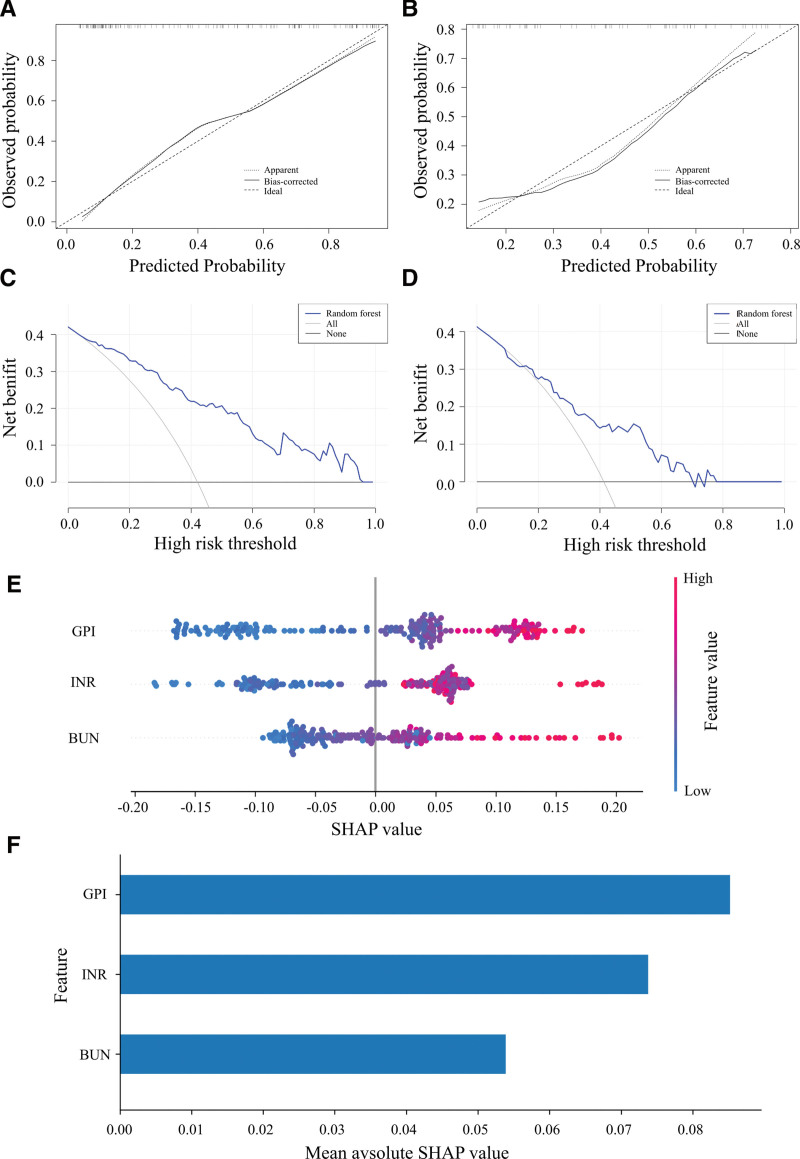
Evaluation of random forest model performance and interpretability. (A and B) Calibration curves of the RF model in the training set (A) and test set (B). (C and D) Decision curves of the RF model in the training set (C) and test set (D). (E) SHAP value plots of the RF model illustrating the impact of GPI, INR, and BUN on model predictions. Color gradients indicate feature values (red = high, blue = low), and the vertical line marks the high-risk threshold. (F) SHAP summary plot ranked by mean absolute SHAP values. BUN = blood urea nitrogen, GPI = globulin-to-platelet index, INR = international normalized ratio, RF = random forest, SHAP = SHapley Additive exPlanations.

An RF model based on GPI, INR, and BUN achieved an AUROC of 0.863 (95% CI, 0.802–0.917) in the training set and 0.747 (95% CI, 0.602–0.863) in the test set (Table 4). Calibration curves based on 1000 bootstrap resamples showed good agreement between predicted and observed outcomes in both the training and test sets, with only minor deviations (Fig. 2A and B). Decision curve analysis demonstrated higher net benefit than treat-all or treat-none strategies across most threshold probabilities (Fig. 2C and D), confirming its clinical utility. SHAP plots illustrated relationships between each RF model variable and the prediction of severe fibrosis in patients with AIH (Fig. 2E). Plots revealed that GPI, INR, and BUN were all risk factors of severe fibrosis, with GPI the most influential feature (highest mean absolute SHAP value) (Fig. 2F).

**Table 4:** Performance of random forest model for discriminating severe fibrosis in the training and test sets.

	Training set	Test set
AUROC (95% CI)	0.863 (0.802–0.917)	0.747 (0.602–0.863)
Sensitivity (95% CI)	0.639 (0.516–0.758)	0.654 (0.458–0.821)
Specificity (95% CI)	0.857 (0.780–0.930)	0.757 (0.615–0.882)
Accuracy (95% CI)	0.766 (0.690–0.834)	0.714 (0.603–0.825)
F1 score (95% CI)	0.696 (0.593–0.783)	0.654 (0.471–0.788)

AUROC = area under the receiver operating characteristic curve, CI = confidence interval.

### 3.4. RF model outperformed APRI and FIB-4 in predicting severe fibrosis

APRI and FIB-4 are noninvasive tools for assessing liver fibrosis. In the entire retrospective cohort, the RF model outperformed APRI (AUROC, 0.557 [95% CI, 0.487–0.643]) and FIB-4 (AUROC, 0.602 [95% CI, 0.525–0.682]), achieving an AUROC of 0.827 (95% CI, 0.770–0.882) (Holm-adjusted *P* < .001 for both comparisons) (see Table S2, Supplemental Digital Content, which shows overall and inflammation-stratified performance of RF, APRI, and FIB-4 for discriminating severe fibrosis).

To evaluate the impact of inflammatory status on model performance, we performed a subgroup analysis. In the G0 to G2 subgroup (n = 103), the RF model maintained high performance (AUROC, 0.842 [95% CI, 0.757–0.914]). In the G3 to G4 subgroup (n = 105), the RF model showed minimal degradation (AUROC, 0.814 [95% CI, 0.726–0.886]). APRI and FIB-4 demonstrated poor diagnostic accuracy across subgroups and deteriorated under moderate-to-severe inflammation: APRI AUROC declined to 0.498 (95% CI, 0.470–0.682), FIB-4 AUROC fell to 0.511 (95% CI, 0.402–0.614) (see Table S2, Supplemental Digital Content).

### 3.5. Integration of TE enhanced RF model performance

**Figure 3.**
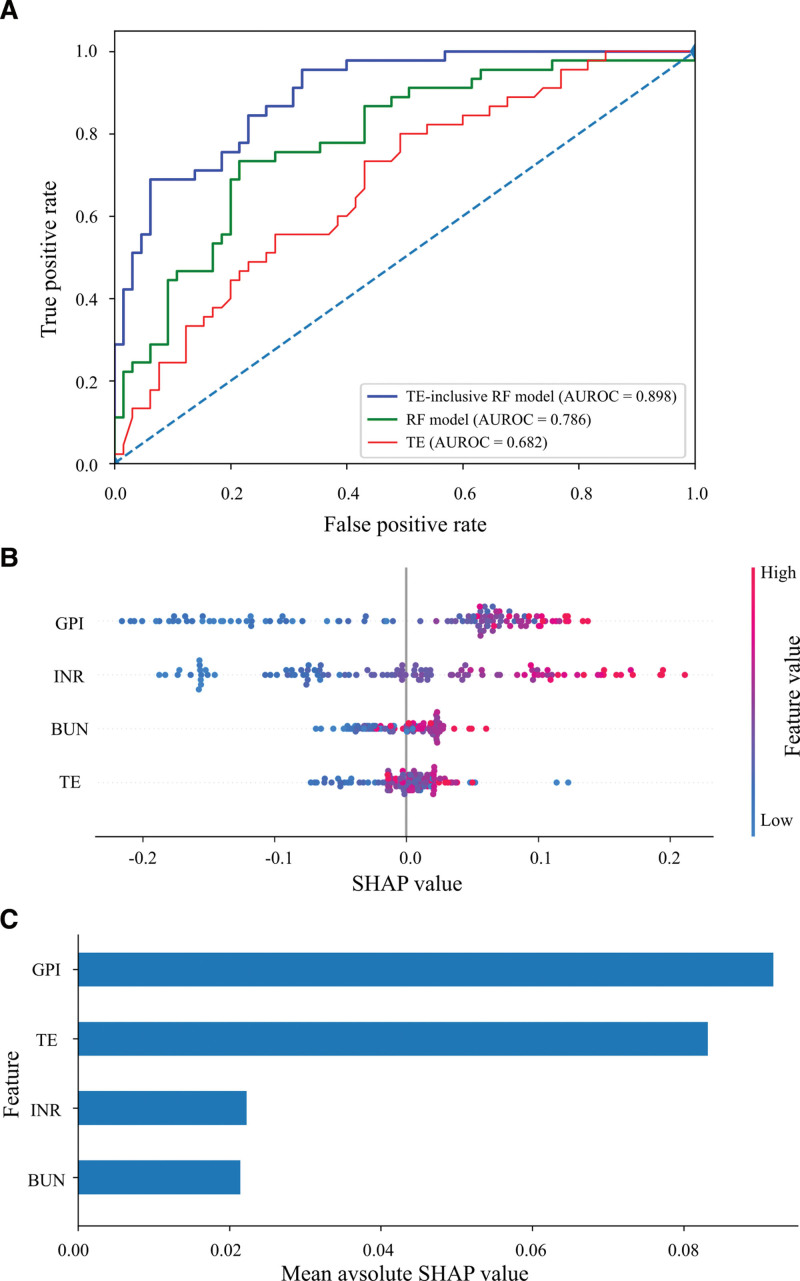
RF outperformed TE in diagnostic accuracy, and the integration of TE into RF enhanced its performance. (A) ROC curves of TE, RF model, and TE-inclusive RF model were conducted to assess their diagnostic performance for severe fibrosis. (B) SHAP value plots of the TE-inclusive RF model illustrating the impact of GPI, INR, BUN, and TE on model predictions. Color gradients indicate feature values (red = high, blue = low), and the vertical line marks the high-risk threshold. (C) SHAP summary plot ranked by mean absolute SHAP values. Abbreviations: AUROC = area under the receiver operating characteristic curve, BUN = blood urea nitrogen, GPI = globulin-to-platelet index, INR = international normalized ratio, RF = random forest, ROC = receiver operating characteristic curve, SHAP = SHapley Additive exPlanations, TE = transient elastography.

Among all patients, 110 underwent LSM with TE. Median LSM was significantly higher in patients with severe fibrosis versus nonsevere fibrosis (*P* = .001). All valid measurements had interquartile-range-to-median ratio <30.0% (see Table S3, Supplemental Digital Content, which compares liver stiffness measurement in patients with severe versus nonsevere fibrosis). In this subgroup, we compared the discriminative ability of the RF model and TE alone for severe liver fibrosis; ROC curves are presented in Figure 3A. Compared with TE, the RF model achieved a higher AUROC (RF, 0.786 [95% CI, 0.699–0.867]; TE, 0.682 [95% CI, 0.575–0.778]). After integrating TE into the RF framework (TE-inclusive RF model), performance improved across metrics: AUROC increased to 0.898 (95% CI, 0.841–0.949), with accuracy 0.818, specificity 0.908, F1 score 0.756, and sensitivity 0.689 (see Table S4, Supplemental Digital Content, which shows performance of RF, TE, and TE-inclusive RF for predicting severe fibrosis in the TE subgroup, stratified by inflammation grade).

Subgroup analysis stratified by inflammation severity revealed slightly lower AUROCs for both the RF model and TE in the G3 to G4 subgroup versus the overall TE subgroup (RF, 0.781 [95% CI, 0.638–0.897] vs 0.786 [95% CI, 0.699–0.867]; TE, 0.672 [95% CI, 0.508–0.816] vs 0.682 [95% CI, 0.575–0.778]). The TE-inclusive RF model maintained robust performance, with a marginally higher AUROC in the G3 to G4 subgroup (0.905 [95% CI, 0.811–0.972] vs 0.898 [95% CI, 0.841–0.949]) (see Table S4, Supplemental Digital Content). Figure 3B displays the SHAP plot for the TE-inclusive RF model. Feature importance ranking (Fig. 3C) confirmed that GPI had the highest mean absolute SHAP value, followed by TE. These results indicate that inclusion of TE enhances predictive capability and stability of the RF model.

### 3.6. RF model retained predictive accuracy in the prospective cohort

**Figure 4.**
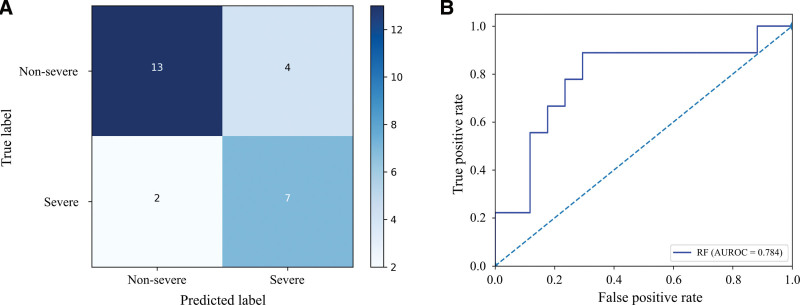
Performance of the RF model in the prospective validation cohort. (A) Confusion matrix showing classification of severe versus nonsevere fibrosis. (B) ROC curve of the RF model in discriminating severe fibrosis. AUROC = area under the receiver operating characteristic curve, RF = random forest, ROC = receiver operating characteristic curve.

To evaluate clinical applicability of the RF model, we prospectively validated its performance for predicting severe fibrosis in an independent cohort of 26 patients. The RF model correctly classified 13 of 17 nonsevere fibrosis cases (true negatives) and 7 of 9 severe fibrosis cases (true positives) (Fig. 4A), yielding a sensitivity of 0.778 and a specificity of 0.765. The model achieved an AUROC of 0.784 (95% CI, 0.556–0.959) in the prospective cohort (Fig. 4B), confirming robust generalizability and clinical utility.

## 4. Discussion

Most patients with AIH have fibrosis or cirrhosis at presentation because of the lack of specific diagnostic markers and delayed recognition.^[27]^ In this study, we identified GPI, INR and BUN as independent predictors of severe liver fibrosis (S3–S4) in AIH. To our knowledge, this is the first study to validate GPI as an independent predictor of fibrosis severity in AIH. An RF model based on these 3 variables showed robust discrimination, preserved performance across inflammatory grades, and acceptable accuracy in prospective validation. Compared with conventional serum scores, the model performed better, and its performance was further enhanced when TE was integrated, supporting a two-tiered deployment strategy: the standalone RF model offers a practical solution where TE is unavailable, while the TE-inclusive model provides enhanced accuracy in resource-rich settings.

GPI integrates immune activation (via globulin) and thrombocytopenia (via platelets). It has been validated as a predictor of liver fibrosis and cirrhosis in CHB patients.^[19,28]^ However, its diagnostic value in AIH has not been previously reported. In our study, the GPI levels were elevated in the severe fibrosis group. This elevation was driven primarily by significantly lower platelet counts (*P* = .002), whereas globulin levels showed no significant difference (*P* = .366). Consistently, univariable logistic regression showed that GPI had a higher absolute coefficient than platelet count alone, supporting its combined utility.

While GPI captures immune-thrombocytopenic interplay, INR reflects hepatic synthetic function, another critical dimension of liver impairment. INR is a commonly used biochemical indicator of cirrhosis, and its elevation can help predict severe liver fibrosis. INR values were higher in patients with AIH and cirrhosis than in those with AIH but without cirrhosis,^[29]^ a trend also observed between severe and nonsevere fibrosis groups in our cohort. Ding et al^[30]^ reported that INR was an independent predictor of advanced fibrosis and cirrhosis in CHB. Their subsequent work confirmed this finding in additional chronic liver disease cohorts.^[31]^ Our findings align with these results, indicating that elevated INR reflects impaired coagulation function associated with progressive liver fibrosis.

As the liver is the primary site of urea synthesis, patients with advanced liver fibrosis or cirrhosis are prone to hepatorenal syndrome and portal hypertension, which can elevate serum urea nitrogen levels.^[32]^ While BUN has shown prognostic value in viral hepatitis and cirrhosis complications,^[33,34]^ its role in AIH specifically remains unclear. In our cohort, BUN contributed independent predictive information in multivariable modeling, although it may be influenced by confounding factors such as diuretic use and dehydration.

Our findings confirmed the limited diagnostic utility of APRI and FIB-4 in AIH, consistent with previous reports.^[14,24,35]^ Their inferior performance likely reflects derivation from viral hepatitis cohorts and documented susceptibility to inflammatory confounding. Notably, while APRI and FIB-4 performance declined in the moderate-to-severe inflammation (G3–G4) subgroup in our study, the RF model maintained relatively stable accuracy across inflammatory subgroups, underscoring its particular advantage in the fluctuating inflammatory context characteristic of AIH.

TE assesses liver stiffness to estimate fibrosis stage, but its accuracy may be compromised by inflammation. In this study, TE demonstrated limited standalone utility in the AIH cohort, likely due to active necroinflammation affecting liver stiffness measurements. This observation aligns with reports that reliable TE assessment may require prolonged immunosuppression in AIH.^[14,36]^ Importantly, rather than replacing TE, the RF model effectively complemented it: integrating TE with serological markers significantly enhanced diagnostic accuracy, suggesting the RF framework can mitigate inflammation-related variability inherent in elastography.

This study has several limitations. First, the single-center design and sample size restrict generalizability, and the model addresses binary classification (severe vs nonsevere) rather than full histologic staging. Second, the TE-inclusive model was developed and evaluated within the same retrospective TE population, which may overestimate performance. Third, residual confounding from treatment status and concomitant medications cannot be excluded. Future studies should include multicenter external validation and prospective impact evaluations.

## 5. Conclusions

GPI, INR, and BUN were identified as independent predictors of severe fibrosis in patients with AIH. An RF model constructed from these 3 markers provided a noninvasive tool for discriminating severe fibrosis that was robust to inflammatory activity. When TE data were available, integration of TE into the model further improved diagnostic performance.

## Author contributions

**Conceptualization:** Haiping Zhang, Jianping Ren, Xinming Li.

**Data curation:** Haiping Zhang, Yinxue Ma, Lijuan Li, Chen Shao.

**Formal analysis:** Haiping Zhang, Jianping Ren, Xinming Li.

**Project administration:** Huiping Yan, Yanmin Liu, Jing Wang.

**Software:** Xinming Li, Kechi Fang.

**Supervision:** Huiping Yan, Yanmin Liu, Jing Wang.

**Validation:** Jianping Ren, Chen Shao, Kechi Fang, Jie Lu.

**Visualization:** Xinming Li.

**Writing – review & editing:** Yinxue Ma, Lijuan Li, Jie Lu, Huiping Yan.

**Writing – original draft:** Haiping Zhang, Jianping Ren, Xinming Li.








