# Supplementary material for: A machine learning model incorporating the globulin-to-platelet index for predicting severe fibrosis in autoimmune hepatitis: A retrospective and prospective validation study
Source: Medicine (Baltimore). 2026 May 8;105(19):e48408. doi: 10.1097/MD.0000000000048408 (PMC13166810; doi:10.1097/MD.0000000000048408)
Supplement: Supplementary file 1 [file medi-105-e48408-s001.docx]

Article title: A Machine Learning Model Incorporating the Globulin-to-Platelet Index for Predicting Severe Fibrosis in Autoimmune Hepatitis: A Retrospective and Prospective Validation Study

First author: Haiping Zhang

**Table S1** Comparison of baseline characteristics between training and test sets in the retrospective cohort

| **Variables** | **Training set (n=145)** | **Test set (n=63)** | ***P* value** |
| --- | --- | --- | --- |
| Female (%) | 125 (86.21) | 57 (90.48) | .392 |
| Age (years) | 55.00 (47.00, 61.00) | 55.00 (49.00, 63.00) | .478 |
| ALT (U/L) | 73.20 (34.00, 127.50) | 70.00 (38.45, 110.50) | .759 |
| AST (U/L) | 96.72 (54.00, 173.30) | 97.00 (62.88, 151.19) | .850 |
| AST/ALT | 1.36 (0.93, 2.20) | 1.44 (0.99, 2.07) | .619 |
| ALP (U/L) | 117.00 (91.00, 152.00) | 119.00 (102.10, 156.00) | .480 |
| GGT (U/L) | 123.00 (60.00, 201.00) | 116.22 (63.50, 191.77) | .710 |
| TP (g/L) | 72.47 ± 7.84 | 73.45 ± 7.33 | .388 |
| Albumin (g/L) | 35.49 ± 4.70 | 35.63 ± 5.51 | .855 |
| Globulin (g/L) | 36.00 (32.10, 41.90) | 37.10 (33.87, 42.30) | .266 |
| A/G | 0.99 ± 0.24 | 0.97 ± 0.23 | .426 |
| INR | 1.11 (1.03, 1.19) | 1.13 (1.04, 1.22) | .506 |
| PT (S) | 11.71 ± 1.87 | 11.96 ± 2.24 | .436 |
| MCH (pg) | 31.50 (30.40, 32.90) | 31.60 (30.50, 32.95) | .915 |
| WBC (*10^9^/L) | 4.83 (3.74, 6.26) | 4.32 (3.63, 5.14) | .051 |
| Hematocrit (%) | 36.23 ± 4.61 | 36.41 ± 5.31 | .817 |
| RBC (*10^12^/L) | 3.86 ± 0.49 | 3.89 ± 0.68 | .807 |
| Monocyte (*10^9^/L) | 0.41 ± 0.15 | 0.40 ± 0.13 | .699 |
| Monocyte% (%) | 8.00 (6.60, 9.80) | 8.60 (7.50, 10.35) | .070 |
| Lymphocyte (*10^9^/L) | 1.69 (1.29, 2.24) | 1.64 (1.22, 2.21) | .511 |
| Lymphocyte% (%) | 36.72 ± 11.90 | 39.00 ± 10.87 | .180 |
| PLT (*10^9^/L) | 152.00 (110.00, 198.00) | 139.00 (105.50, 193.50) | .514 |
| eGFR (mL/min/1.73m^2^) | 106.70 (99.90, 112.72) | 106.96 (100.28, 113.80) | .637 |
| Creatinine (μmol/L) | 50.72 (44.00, 57.20) | 48.60 (44.00, 52.61) | .129 |
| BUN (mmol/L) | 4.30 (3.67, 4.98) | 4.04 (3.59, 4.64) | .188 |
| DB (μmol/L) | 18.10 (8.20, 31.90) | 17.90 (8.45, 36.25) | .531 |
| TB (μmol/L) | 29.90 (20.26, 47.70) | 32.20 (22.15, 55.85) | .273 |
| Immunoglobulin A (g/L) | 3.29 (2.78, 4.10) | 3.70 (3.02, 4.34) | .103 |
| Immunoglobulin M (g/L) | 1.29 (0.97, 1.63) | 1.23 (0.98, 1.63) | .983 |
| Immunoglobulin G (g/L) | 21.12 (18.58, 24.40) | 21.44 (18.89, 24.44) | .643 |
| GPI | 2.43 (1.81, 3.43) | 2.72 (1.94, 3.73) | .283 |
| APRI | 1.64 (0.82, 2.90) | 1.81 (0.89, 2.88) | .624 |
| FIB-4 | 3.86 (2.35, 7.54) | 4.65 (2.68, 8.93) | .325 |
| ANA (+) | 142/145 (97.93) | 60/62 (96.77) | .998 |
| ASMA (+) | 38/145 (26.21) | 11/62 (17.74) | .189 |
| anti-SLA/LP (+) | 10/139 (7.19) | 4/60 (6.67) | 1.000 |
| anti-LC-1 (+) | 0/145 (0.00) | 1/59 (1.69) | .296 |
| anti-LKM-1 (+) | 1/145 (0.69) | 0/62 (0.00) | 1.000 |
| Fibrosis stage |  |  | .935 |
| S0-S1 | 30 (20.69) | 11 (17.46) |  |
| S2 | 54 (37.24) | 26 (41.27) |  |
| S3 | 40 (27.59) | 17 (26.98) |  |
| S4 | 21 (14.48) | 9 (14.29) |  |
| Complications |  |  |  |
| Liver cirrhosis | 17 (11.72) | 5 (7.94) | .414 |
| Ascites | 8 (5.52) | 2 (3.17) | .709 |
| Hepatic Encephalopathy | 0 (0.00) | 2 (3.17) | .091 |
| Intra-abdominal Infection | 1 (0.69) | 0 (0.00) | 1.000 |
| Inflammatory grade | |  | .809 |
| G0–G2 | 71 (48.97) | 32 (50.79) |  |
| G3–G4 | 74 (51.03) | 31 (49.21) |  |

Abbreviations: A/G = albumin-to-globulin ratio; ALP = alkaline phosphatase; ALT = alanine aminotransferase; ANA = anti-nuclear antibody; anti-LC-1 = anti-liver cytosol type 1 antibody; anti-LKM-1 = anti-liver kidney microsomal type 1 antibody; anti-SLA/LP = anti-soluble liver antigen/liver-pancreas antigen antibody; APRI = aspartate aminotransferase-to-platelet ratio index; ASMA = anti-smooth muscle antibody; AST = aspartate aminotransferase; BUN = blood urea nitrogen; DB = direct bilirubin; FIB-4 = fibrosis-4 index; eGFR = estimated glomerular filtration rate; GGT = gamma-glutamyl transferase; GPI = globulin-to-platelet index; INR = international normalized ratio; MCH = mean corpuscular hemoglobin; PLT = platelet count; PT = prothrombin time; RBC = red blood cell count; TB = total bilirubin; TP = total protein; WBC = white blood cell count.
