# Supplementary material for: A machine learning model incorporating the globulin-to-platelet index for predicting severe fibrosis in autoimmune hepatitis: A retrospective and prospective validation study
Source: Medicine (Baltimore). 2026 May 8;105(19):e48408. doi: 10.1097/MD.0000000000048408 (PMC13166810; doi:10.1097/MD.0000000000048408)
Supplement: Supplementary file 2 [file medi-105-e48408-s002.docx]

Article title: A Machine Learning Model Incorporating the Globulin-to-Platelet Index for Predicting Severe Fibrosis in Autoimmune Hepatitis: A Retrospective and Prospective Validation Study

First author: Haiping Zhang

**Table S2** Performance of RF, APRI and FIB4 for discriminating severe fibrosis by inflammation grade

|  | **Total**  **(n = 208)** | **G0-G2**  **(n = 103)** | **G3-G4**  **(n = 105)** |
| --- | --- | --- | --- |
| RF |  |  |  |
| AUROC (95% CI) | 0.827 (0.770-0.882) | 0.842 (0.757-0.914) | 0.814 (0.726-0.886) |
| Cutoff value (95% CI) | 0.500 | 0.500 | 0.500 |
| Sensitivity (95% CI) | 0.644 (0.545-0.740) | 0.564 (0.400-0.732) | 0.708 (0.571-0.826) |
| Specificity (95% CI) | 0.826 (0.758-0.892) | 0.859 (0.765-0.939) | 0.789 (0.679-0.889) |
| Accuracy (95% CI) | 0.75 (0.688-0.808) | 0.748 (0.670-0.835) | 0.752 (0.667-0.819) |
| F1-score (95% CI) | 0.683 (0.600-0.759) | 0.629 (0.483-0.753) | 0.723 (0.615-0.808) |
| APRI |  |  |  |
| AUROC (95% CI) | 0.557 (0.487-0.643) | 0.607 (0.405-0.653) | 0.498 (0.470-0.682) |
| Cutoff value (95% CI) | 1.537 | 1.537 | 1.537 |
| Sensitivity (95% CI) | 0.69 (0.587-0.790) | 0.641 (0.474-0.795) | 0.729 (0.595-0.857) |
| Specificity (95% CI) | 0.463 (0.373-0.552) | 0.562 (0.433-0.690) | 0.351 (0.222-0.469) |
| Accuracy (95% CI) | 0.558 (0.490-0.625) | 0.592 (0.495-0.689) | 0.524 (0.429-0.610) |
| F1-score (95% CI) | 0.566 (0.487-0.643) | 0.543 (0.405-0.653) | 0.583 (0.470-0.682) |
| *P* value of AUROC  compared to RF | <.001 | <.001 | <.001 |
| FIB-4 |  |  |  |
| AUROC (95% CI) | 0.602 (0.525-0.682) | 0.682 (0.568-0.785) | 0.511 (0.402-0.614) |
| Cutoff value (95% CI) | 5.095 | 5.095 | 5.095 |
| Sensitivity (95% CI) | 0.529 (0.430-0.634) | 0.513 (0.349-0.675) | 0.542 (0.405-0.682) |
| Specificity (95% CI) | 0.62 (0.527-0.706) | 0.719 (0.606-0.831) | 0.509 (0.380-0.627) |
| Accuracy (95% CI) | 0.582 (0.514-0.649) | 0.641 (0.544-0.738) | 0.524 (0.438-0.610) |
| F1-score (95% CI) | 0.514 (0.429-0.604) | 0.519 (0.368-0.651) | 0.51 (0.382-0.619) |
| *P* value of AUROC  compared to RF | <.001 | .002 | <.001 |

Abbreviations: APRI = aspartate aminotransferase-to-platelet ratio index, AUROC = area under the receiver operating characteristic curve, FIB-4 = fibrosis-4 index, RF = random forest.
