# Supplementary material for: A machine learning model incorporating the globulin-to-platelet index for predicting severe fibrosis in autoimmune hepatitis: A retrospective and prospective validation study
Source: Medicine (Baltimore). 2026 May 8;105(19):e48408. doi: 10.1097/MD.0000000000048408 (PMC13166810; doi:10.1097/MD.0000000000048408)
Supplement: Supplementary file 3 [file medi-105-e48408-s003.docx]

Article title: A Machine Learning Model Incorporating the Globulin-to-Platelet Index for Predicting Severe Fibrosis in Autoimmune Hepatitis: A Retrospective and Prospective Validation Study

First author: Haiping Zhang

**Table S3** Comparison of liver stiffness measurement in patients with severe and nonsevere fibrosis

| **Variables** | **Total (n = 110)** | **Nonsevere fibrosis (n = 65)** | **Severe fibrosis (n = 45)** | ***P* value** |
| --- | --- | --- | --- | --- |
| LSM (kPa) | 20.550 (14.250, 28.925) | 17.900 (12.800, 24.850) | 24.500 (18.550, 36.200) | .001 |
| IQR (kPa) | 2.900 (1.600, 4.800) | 2.600 (1.300, 3.900) | 4.100 (1.900, 7.100) | .003 |
| IQR/median (%) | 15.000 (9.000, 22.000) | 15.000 (9.000, 21.500) | 16.000 (10.000, 23.000) | .382 |

Abbreviations: IQR = interquartile range, IQR/median = interquartile range-to-median ratio, LSM = liver stiffness measurement.
