# Supplementary material for: A machine learning model incorporating the globulin-to-platelet index for predicting severe fibrosis in autoimmune hepatitis: A retrospective and prospective validation study
Source: Medicine (Baltimore). 2026 May 8;105(19):e48408. doi: 10.1097/MD.0000000000048408 (PMC13166810; doi:10.1097/MD.0000000000048408)
Supplement: Supplementary file 4 [file medi-105-e48408-s004.docx]

Article title: A Machine Learning Model Incorporating the Globulin-to-Platelet Index for Predicting Severe Fibrosis in Autoimmune Hepatitis: A Retrospective and Prospective Validation Study

First author: Haiping Zhang

**Table S4** Performance of RF, TE and TE-inclusive RF for predicting severe fibrosis by inflammation grade

|  | **Total (n = 110)** | **G0-G2 (n = 61)** | **G3-G4 (n = 49)** |
| --- | --- | --- | --- |
| RF |  |  |  |
| AUROC (95% CI) | 0.786 (0.699-0.867) | 0.793 (0.659-0.900) | 0.781 (0.638-0.897) |
| Cutoff value (95% CI) | 0.500 | 0.500 | 0.500 |
| Sensitivity (95% CI) | 0.622 (0.487-0.745) | 0.591 (0.381-0.790) | 0.652 (0.450-0.833) |
| Specificity (95% CI) | 0.800 (0.701-0.891) | 0.795 (0.659-0.912) | 0.808 (0.645-0.955) |
| Accuracy (95% CI) | 0.727 (0.645-0.800) | 0.721 (0.607-0.820) | 0.735 (0.612-0.857) |
| F1-score (95% CI) | 0.651 (0.524-0.755) | 0.605 (0.410-0.764) | 0.698 (0.516-0.837) |
| TE |  |  |  |
| AUROC (95% CI) | 0.682 (0.575-0.778) | 0.675 (0.526-0.790) | 0.672 (0.508-0.816) |
| Cutoff value (95% CI) | 19.300 | 19.300 | 19.300 |
| Sensitivity (95% CI) | 0.733 (0.588-0.864) | 0.682 (0.500-0.864) | 0.783 (0.600-0.944) |
| Specificity (95% CI) | 0.569 (0.451-0.696) | 0.615 (0.465-0.758) | 0.500 (0.292-0.680) |
| Accuracy (95% CI) | 0.636 (0.536-0.727) | 0.639 (0.508-0.738) | 0.633 (0.490-0.755) |
| F1-score (95% CI) | 0.623 (0.505-0.727) | 0.577 (0.400-0.720) | 0.667 (0.500-0.793) |
| *P* value of AUROC  compared to RF | <.001 | <.001 | <.001 |
| TE-inclusive RF |  |  |  |
| AUROC (95% CI) | 0.898 (0.841-0.949) | 0.890 (0.802-0.955) | 0.905 (0.811-0.972) |
| Cutoff value (95% CI) | 0.500 | 0.500 | 0.500 |
| Sensitivity (95% CI) | 0.689 (0.553-0.816) | 0.636 (0.421-0.842) | 0.739 (0.550-0.889) |
| Specificity (95% CI) | 0.908 (0.829-0.970) | 0.897 (0.794-0.976) | 0.923 (0.793-1.000) |
| Accuracy (95% CI) | 0.818 (0.745-0.891) | 0.803 (0.705-0.886) | 0.837 (0.714-0.918) |
| F1-score (95% CI) | 0.756 (0.642-0.854) | 0.700 (0.514-0.844) | 0.810 (0.649-0.909) |
| *P* value of AUROC  compared to RF | <.001 | .038 | .016 |

Abbreviations: AUROC = area under the receiver operating characteristic curve, CI = confidence interval, RF = random forest, TE = transient elastography.
